# Relationships of work stress and interpersonal needs with industrial workers’ mental health: a moderated mediation model

**DOI:** 10.1186/s12889-023-16002-1

**Published:** 2023-07-12

**Authors:** Rongxi Wang, Yujie Liu, Shangbin Liu, Hui Chen, Xiaoyue Yu, Chen Xu, Yingjie Chen, Danni Xia, Xin Ge, Zhiqiang Wang, Ruijie Chang, Fan hu, Tian Shen, Ying Wang, Zixin Wang, Bolin Cao, Kechun Zhang, Huachun Zou, Jiade Qin, Sisi Li, Yong Cai

**Affiliations:** 1 Shanghai University of Medicine & Health Sciences Affiliated Zhoupu Hospital, 1500 Zhouyuan Road, Shanghai, 201318 China; 2 School of Public Health, Shanghai Jiao Tong University School of Medicine, South Chongqing Road, No. 227, Shanghai, 201800 China; 3 JC School of Public Health and Primary Care, Faculty of Medicine, The Chinese University of Hong Kong, Hong Kong, 999077 China; 4 School of Media and Communication, Shenzhen University, Shenzhen, 518000 China; 5 Shenzhen Longhua District Center for Disease Control and Prevention, Shenzhen, China; 6 School of Public Health (Shenzhen), SunYat-sen University, Shenzhen, 518107 China; 7 Kirby Institute, University of New South Wales, Sydney, 2052 Australia; 8 The First People’s Hospital of Qinzhou; The Tenth Affiliated Hospital of Guangxi Medical University, Yangming Street, No. 8, Qinzhou, Guangxi Province 535099 China

**Keywords:** Depression, Anxiety, Defeat, Work stress, Interpersonal needs, Industrial workers

## Abstract

**Objectives:**

This study explores whether feelings of defeat (i.e., a sense of failed struggle and losing rank; referred to as defeat for simplicity) mediated the effect of work stress on depression/anxiety, the effect of interpersonal needs on depression/anxiety for Chinese industrial workers, and the possible moderating role of social support.

**Method:**

A cross-sectional study was conducted in Shenzhen, China in 2019, in total, 2023 industrial workers (of 2700 invited; response rate = 75%) completed a self-administered survey consisted of Job Stress Scale, Interpersonal Needs Questionnaire, Defeat Scale, Centre for Epidemiological Studies Depression Scale, Generalized Anxiety Disorder Scale, two face-valid questions for social support, as well as sociodemographic information. Moderated mediation model was tested and loop plots were applied to probe into the conditional effects of work and interpersonal stress on depression and anxiety symptoms.

**Result:**

Both the direct and indirect effect of work stress on depression and anxiety through defeat were significant (Work stress→ Depression: *B* = 0.035, *p* < .001, Work stress→ Defeat→ Depression: *B* = 0.034, *p* < .001; Work stress→ Anxiety: *B* = 0.038, *p* < .001, Work stress→ Defeat→ Anxiety: *B* = 0.045, *p* < .001). Meanwhile, defeat mediated the relationship of interpersonal needs with depression partially and the relationship of interpersonal needs with anxiety totally (Interpersonal needs→ Anxiety: B = 0.133, p < .001, Interpersonal needs→ Defeat→ Anxiety: *B* = 0.010, *p* = .537). Social support moderated the indirect path between interpersonal needs and depression/anxiety and buffered the effect.

**Conclusion:**

The mediating role of defeat and the moderator role of social support in the relationship between stress and depression/anxiety were confirmed in industrial workers. Workers who reported more work and interpersonal stress would report more defeat feelings, and then exhibited more depression and anxiety symptoms; this mediation effect was stronger for those who had lower social support, respectively.

**Supplementary Information:**

The online version contains supplementary material available at 10.1186/s12889-023-16002-1.

## Introduction

Depression and anxiety were identified as leading problems of workplace mental health for industrial workers [1]. Using validated self-report questionnaires or interview assessments, studies reported that the detection rates of depression and anxiety for worker populations could be as high as 50% among industrial workers [2]. The prevalence of clinically relevant depressive symptoms was 38.6% among industrial workers in Vietnam and 28% among migrant workers in China [3, 4]. Frequently observed mental health issues included, but not limited to, life dissatisfaction, sleep disorder, depression, anxiety, and even suicide attempts [5, 6]. Along with elevated risks for subsequent health issues [7], organizational labor costs, and socioeconomic stability concern, mental health issue of Chinese industrial workers has become more and more important. Substantial co-morbidity has often been observed with one-third to one-half of participants with depression also reporting having a history of anxiety [8].However, joint underlying mechanisms were largely overlooked in previous studies. Moreover, work-associated psychiatric disorders could be influenced by not only workplace characteristics but also individual susceptibilities, while the latter did not receive enough attention in previous research. The current study was designed in response to these issues.

### Work stress and interpersonal needs in industrial workers

Work stress, namely the harmful physical and emotional responses that occur when the requirements of the job do not match the capabilities, resources, or needs of the worker, is one of the main workplace stressors [9]. Nearly 41-83% of workers are exposed to work stress, and these rates seem to have increased during the last decades because of intensified workloads, high job insecurity, and low job quality [10, 11]. The prevalence of psychiatric morbidity was greater in high job-strain individuals, and the prevalence of severe anxiety, worry, and fatigue symptoms was significantly greater in such a population after adjusting for socioeconomic factors [12]. A cohort study in New Zealand showed that participants exposed to high work stress (viz., excessive workload, extreme time pressures) had a twofold risk of major depressive disorder or generalized anxiety disorder compared to those with low work stress [2]. The demand-control model proposed that adverse health effects are expected when workers were exposed to high psychological demands in combination with low decision latitude [13]. The effort-reward-imbalance model suggested that mental distress and its health correlates arise when a high degree of effort was not adequately rewarded in the form of pay, esteem, status consistency, or career opportunities [14]. Both theories share the notion that poor work conditions, especially excessive occupational stress, have a detrimental effect on mental health.

Interpersonal needs, namely the mental state of thwarted belongingness and perceived burdensomeness, reflect chronically unfulfilled social competence and belonging needs [15, 16]. The associations between unmet interpersonal needs and depression and anxiety were well documented [17–19]. Industrial workers could be typically vulnerable to unmet interpersonal needs, as they usually migrate from low-income cities to central, modernized cities to seek work opportunities, and be apart from their families and friends. Common by-hour or by-piece payment schemes motivate “work more to gain more”, which might add more constraints to social interaction. Typically, Chinese industrial workers are found to engage in relatively fewer social activities, leading to limited social network and increased social isolation [20].

### The mediation effect of defeat

The current investigation proposed that defeat might play an important role between industrial workers’ stressors and depression and anxiety. Defeat refers to the perceptions of failed struggle and low social rank, which has played a core role in evolutionary accounts of depression, anxiety, and suicidality in various populations [21, 22]. Animal models have identified social defeat-associated depression-like behaviours such as reductions in locomotion, decreased motivation and lack of interest in rewarding stimuli in animals, mirroring psychopathological responses in humans [23, 24]. Socially defeated animals adopt short-term protective strategies, including social withdrawal, decreased sleep, feeding, and hypervigilance to dangerous situations, while others became more alert to the external environment with elevated levels of stress hormone [23]. Human studies supported this notion. Gilbert and Allan, who designed the Defeat Scale (DS) to capture a sense of failed struggle and losing rank, argued that defeat was significantly correlated with depression [25]. A longitudinal study showed that baseline levels of defeat predicted 12-month changes in depression and anxiety [26]. By comparing income vs. income rank, Wood and colleagues [27] concluded that the concern for social rank was the mechanism through which income related to distress.

**Fig. 1:**
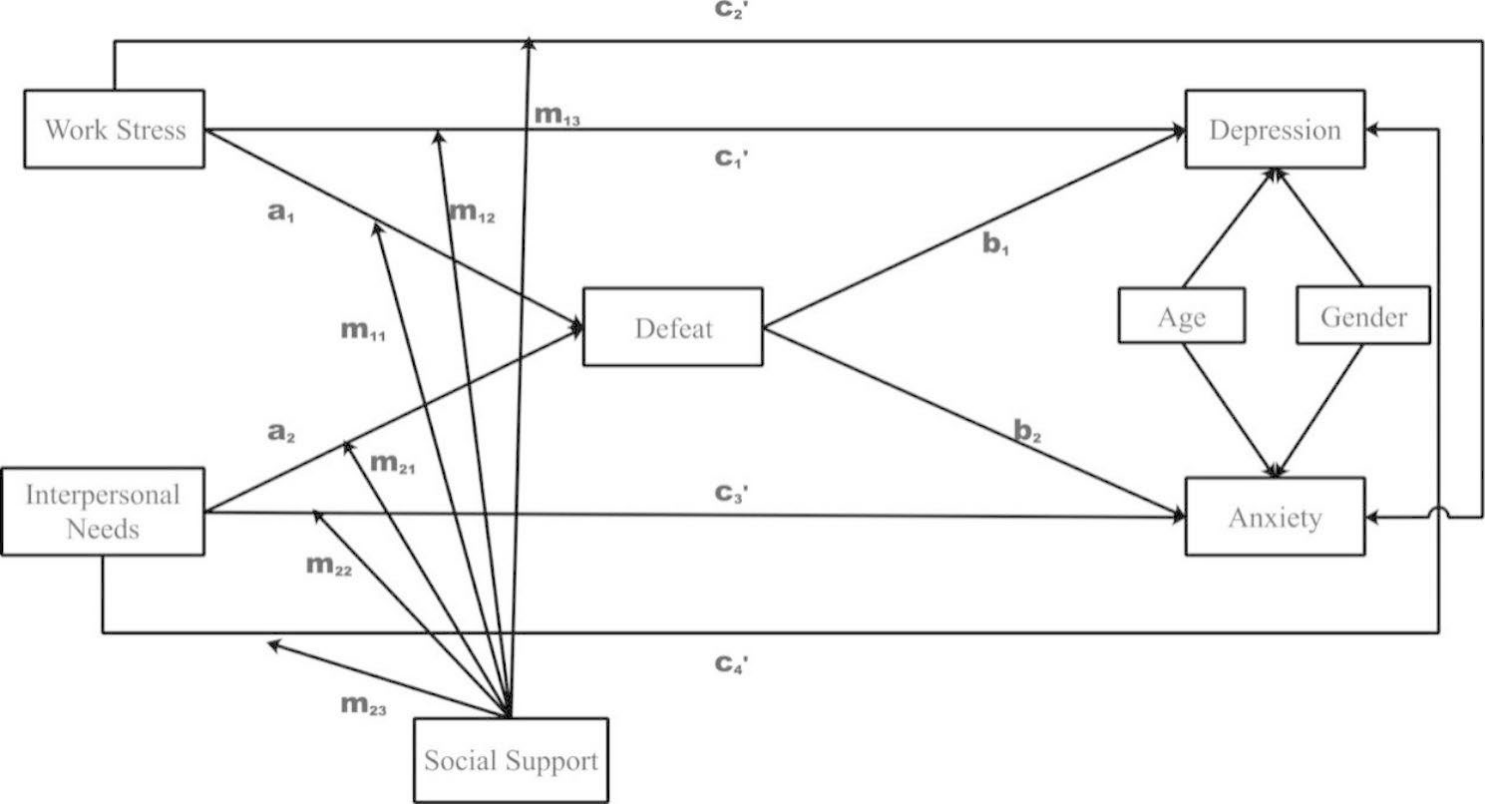
The proposed moderated mediation model ***Note***. Paths **a**_1_ and **a**_**2**_ represent the effects of the independent variables (i.e., work stress and interpersonal needs, respectively) on the mediator (i.e., defeat); paths **b**_**1**_ and **b**_**2**_ represent the effects of the mediator on the dependent variables (i.e., depression and anxiety, respectively); paths **c**_**1**_**’** to **c**_**4**_**’** represent the direct effects of each independent variable on each dependent variable; and paths **m**_**11**_ to **m**_**23**_ represent the moderation effects of social support. Therefore, effects **a**_**1**_ × **b**_**1**_ and **a**_**1**_ × **b**_**2**_ represent the indirect effects of work stress on depression and anxiety via defeat, respectively, and similarly, effects **a**_**2**_ × **b**_**1**_ and **a**_**2**_ × **b**_**2**_ represent the indirect effects from interpersonal needs to depression and anxiety via defeat, respectively. Age and gender were included as the control variables

The drastic industrialization and urbanization in China over the past decades have fueled the boom of migrant, industrial workers, especially in its southern, modern cities. However, previous research revealed that Chinese industrial workers often suffered from stigmatization such as labeling, stereotyping, and status loss [28], or living a marginalized life [29]. Most importantly, they could suffer from the risk of heavy workload and blockage in the fulfilment of basic psychological needs such as autonomy [30], and display less social competence [31]. These work and social stressors might lead to feelings of defeat and subsequently propagate symptoms of depression and anxiety. Therefore, the current study hypothesized that feelings of defeat caused by (heavy) work stress and (thwarted) interpersonal needs might account for depression (*Hypothesis 1.1*, indirect effects represented by **a**_**1**_ × **b**_**1**_ and **a**_**1**_ × **b**_**2**_ in Fig. 1) and anxiety (*Hypothesis 1.2*, indirect effects represented by **a**_**2**_ × **b**_**1**_ and **a**_**2**_ × **b**_**2**_ in Fig. 1) symptoms for Chinese industrial workers; .

### Moderation effect(s) of social support

Social environment characteristics play vital roles in stress coping [32]. A supportive social environment helps protect against excessive, unnecessary neuroendocrine activation, therefore avoiding adverse effects of stress, while the lack thereof could elicit hypothalamic–pituitary–adrenal axis and sympathetic nervous system hyperactivities [33]. The current study further investigated how social support may cast on the linkage between work stress and interpersonal needs to feelings of defeat and depression and anxiety symptoms in Chinese industrial workers. Social support, the perception that one has available assistance, the actually received assistance, or the degree to which a person is integrated into a social network, can not only lower the perception of stress but also buffer the deleterious effect of it [34–37]. Studies revealed that supportive supervisors (e.g., via encouraging autonomy) can motivate workers to worship work efficiency and productivity, and the successful management of job demands help workers remain positive [38]. Furthermore, positive feedbacks from others in the interpersonal process offer constructive effect on one’s emotion and protect against the poor mental state [39]. Therefore, this study hypothesized that social support might not only directly protect against depression and anxiety symptoms from work stress and unmet interpersonal needs (*Hypothesis 2.1*; paths **m**_**12**_, **m**_**13**_ and **m**_**22**_, **m**_**23**_; referring to Fig. 1), but also indirectly mitigate the associated negative effects of via feelings of defeat (*Hypothesis 2.2*; paths **m**_**11**_ and **m**_**21**_; referring to Fig. 1).

Thus, the purpose of this study was to probe the mediating role of defeat in the relationship between work stress and depression/anxiety and the relationship between interpersonal needs and depression/anxiety from a social rank perspective, as well as the moderating role of social support as a critical social environmental characteristic in a sample of Chinese industrial workers who work in industrial factories in Shenzhen.

## Method

### Participants

A cross-sectional survey of industrial workers in Longhua District, Shenzhen (one of China’s largest migrant cities) was conducted from October to December 2019. There were 517,000 fully employed workers and 81.11% of them engaged in manufacturing industry in Longhua District in 2018 [40]. Eligible participants were (1) aged 18 or above; and (2) working full-time in local factories. Assuming 50% of industrial workers experienced excess work stress or unmet interpersonal needs and an error for sampling of 0.05, a sample size of 1889 was required to allow for a non-response rate of 20%.

According to the report of Shenzhen Statistical Yearbook, 9.2%, 48.4%, 3.7%, 0.8%, 0.6%, 4.3%, and 0.4% of the manufacturing industrial workers in Longhua District engaged in machinery processing industry, electronic device manufacturing industry, printing and dyeing industry, chemical material industry, melting industry and garment industry [40]. Combined with the required sample size of the present study, the number of workers to be sampled from each industry was determined prior to data collection. Based on the size of factories, 16 factories, including 4 machinery processing factories, 3 electronic device manufacturing factories, 3 printing and dyeing factories, 2 chemical material factories, 1 melting factory, 1 garment factory, 1 food and beverage manufacturing factory, and 1 other factory were randomly selected. Then, three to four workshops were then randomly selected from each factory. All workers who met the inclusion criteria were invited to participate in a survey conducted by the Center for Disease Control and Prevention, Longhua District.

### Procedure

The study was approved by the Ethics Committee of the School of Public Health, Sun Yat-sen University (2019/3) and conducted under the guidelines of the Declaration of Helsinki. A group of two public health researchers, an epidemiologist, a health psychologist, a health communication expert, and a factory worker designed the survey. A pilot study (*n* = 20; not included in the following analyses) was conducted to validate the psychometric characteristics of the survey. Trained fieldworkers introduced the study’s purposes and obtained consent from the participants, who later completed the self-administered survey in single rooms to ensure privacy. Each participant received a 20-yuan (about 2.60 US dollars, compatible with Shenzhen industrial workers’ hourly minimum payment in 2018 [41]) cash voucher as a reward.

### Materials

*Work Stress.* Work stress was measured by the 13-item Job Stress Scale (e.g., “Are you often unclear about where your job responsibilities lie?”; 1 = *never* to 7 = *always*) [42] which contained four factors, namely, workload, role conflict, role ambiguity and utilization of skills. It had been applied in Chinese culture and exhibited good reliability and validity [43].

*Social Support.* Social support was measured by two items, emotional and instrumental (e.g., “How much support can your family and friends or colleagues give you when you need material help (e.g., financial difficulties)?”; 0 = *never* to 10 = *very much*).

*Interpersonal Needs.* Individuals’ interpersonal needs were measured by the 15-item Interpersonal Needs Questionnaire (INQ) [44]. Participants reported compliance with these scenarios (e.g., “These days, I think I am a burden on society”; 1 = *not true for me at all* to 7 *very true for me*) in the past week. The scale shown good psychometric properties in Chinese workers [45].

*Defeat.* The defeat was measured by the Defeat Scale, a self-report measure of 16 questions (e.g., “I feel defeated by life”; 0 = *never* to 4 = *always*) assessing individuals’ perceptions of losing rank position and failed struggle during the past seven days [25]. Higher scores indicated feelings of more defeat. A previous study found the Chinese version of DS valid and reliable among medical students [46].

*Depression*. The Centre for Epidemiological Studies Depression Scale (CES-D-10) was a short self-reported scale assessing depressive symptoms in the general population [47]. Participants rate how often they have experienced certain feelings during the past week (e.g., *“Sleep disturbance” and “Feeling lonely”*; 0 = *less than a day or never* to 4 = *5–7 days*). The Chinese version of CESD has been applied to measure depression among various populations including old people and factory workers [48, 49].

*Anxiety*. The Generalized Anxiety Disorder (GAD) Scale was applied to measure how often the individuals were bothered by each anxiety symptom during the past two weeks [50]. It is a 7-item scale (e.g., “Feeling nervous, anxious, or on edge”; 1 = *not at all* to 4 = *nearly every day*). The Chinese version of GAD has been validated among a group of pregnant women [51] and applied to adolescents and migrant workers [52, 53].

Finally, participants reported their sociodemographic information, including age, gender, place of origin, marital status, education level, monthly income, living alone or not, weekly working time, and the duration participants worked as migrant workers in Shenzhen (see Supplementary Table 1).

#### Analytical scheme

Data entry of complete responses was conducted by two research assistants using Epidata (The EpiData Association, Odense, Denmark). IBM SPSS Statistics 24.0 (IBM Corp., Armonk, NY, USA) was used to assess the descriptive statistics, psychometric characteristics, and bivariate correlations. Means and standard deviations (SD) are reported for continuous variables and frequency and percentage are reported for categorical variables. Sociodemographic factors associated with depression and anxiety were screened by multiple linear regression. Path analysis using Mplus 8.0 (Muthén & Muthén, Los Angeles, CA, USA) was performed to examine the hypothesized relationships among work stress, interpersonal needs, social support, depression, and anxiety. The mean scores of the summed score of the scales were calculated and analyzed as continuous variables. Bootstrapping analysis with 10,000 resamples was conducted to test the significance of the mediation and moderation effects [54] using ML estimation. Model fit was evaluated through the root mean square error of approximation (RMSEA), comparative fit index (CFI), Tucker–Lewis index (TLI), and standardized root mean square residual (SRMR) were calculated to assess the goodness of model fit. CFI and TLI values > 0.90 and RMSEA and SRMR values < 0.08 were considered as indicating an acceptable model fit [55].

## Results

Totally, 2700 industrial workers were invited to participate and 2023 workers (i.e., response rate = 75%) completed the questionnaire. Sixteen participants were further excluded due to reporting an illogical age value (e.g., “123”), resulting in a final sample of 2007 workers. Sociodemographic characteristics were summarized in Supplementary Table 1. Preliminary analyses suggested only age and gender predicted key outcomes, while education level, marital status, and income were irrelevant. Mean and standardized deviation, Cronbach’s Alpha, and bi-variate correlations were reported in Table 1. A Cronbach’s α with a value equal to or greater than 0.8 indicated acceptable reliability.

**Table 1:** Descriptive statistics, Cronbach’s *α*s and the correlation matrix of key variables

		*α*	*M*	*SD*	1	2	3	4	5	6
1.	Work stress	0.899	35.101	15.310	—					
2.	Interpersonal needs	0.863	34.667	13.950	0.241^*^	—				
3.	Social support	0.813	25.317	10.314	− 0.144^*^	− 0.010	—			
4.	Feelings of defeat	0.925	12.337	10.872	0.273^*^	− 0.138^*^	0.451^*^	—		
5.	Depression	0.829	18.107	5.444	0.199^*^	− 0.113^*^	0.290^*^	0.479^*^	—	
6.	Anxiety	0.918	10.205	4.167	0.211^*^	− 0.153^*^	0.261^*^	0.537^*^	0.605^*^	—

***Note***. *α* = Cronbach’s alpha, Mean = mean, SD = standard deviation. ^*^p < .05

Path analysis examined whether defeat mediated the deleterious effects of work stress and (unmet) interpersonal needs on depression and anxiety, controlling for age and gender. Data fitted the proposed model well (χ^2^ (2) = 17.263, *p* < .001; RMSEA = 0.062; CFI = 0.993; TLI = 0.951; SRMR = 0.021). Depression and anxiety symptoms were slightly correlated (*r* = .106, *p* < .001). Females scored higher than males on both symptoms (*B*_depression_ = 0.106, *SE* = 0.023, 95%CI = [0.061, 0.150], *B*_anxiety_ = 0.103, *SE* = 0.024, 95% CI = [0.054, 0.149]; *p*s < 0.001). Age was negatively associated with both symptoms (*B*_depression_ = -0.005, *SE* = 0.001, 95%CI = [-0.008, -0.003], *B*_anxiety_ = -0.007, *SE* = 0.001, 95% CI = [-0.010, -0.004]; *p*s < 0.001). Results (see Table 2) supported Hypothesis 1.1 such that more work stress elicited more defeat, which then contributed to more depression (i.e., effect **a**_**1**_ × **b**_**1**_) and anxiety (i.e., effect **a**_**1**_ × **b**_**2**_**)** symptoms. Similarly, Hypothesis 1.2 was supported such that more unfulfilled interpersonal needs also elicited more defeat, which then contributed to more depression (i.e., effect **a**_**2**_ × **b**_**1**_) and anxiety (i.e., effect **a**_**2**_ × **b**_**2**_**)** symptoms. Work stress and interpersonal needs explained 23.2% (*p* < .001) of the total variance of defeat. In total, the proposed mediation model explained substantial amounts of the total variances of depression and anxiety (${R}_{depression}^{2}$= 0.252 and ${R}_{anxiety}^{2}$= 0.306; *p*s < 0.001), respectively.

**Table 2:** Results of the mediation model

(Outcome)	Estimate	*S.E.*	*p*	Bootstrap 95% CI
Work stress → Depression				
Total effect	0.069	0.012	< 0.001	[0.046, 0.091]
Indirect effect (paths ${\varvec{a}}_{1}\times {\varvec{b}}_{1}$)	0.034	0.005	< 0.001	[0.025, 0.044]
Direct effect (path ${\varvec{c}}_{1}^{\varvec{{\prime }}}$)	0.035	0.011	0.001	[0.014, 0.056]
Work stress → Anxiety				
Total effect	0.083	0.012	< 0.001	[0.053, 0.106]
Indirect effect (paths ${\varvec{a}}_{1}\times {\varvec{b}}_{2}$)	0.045	0.007	< 0.001	[0.033, 0.058]
Direct effect (path ${\varvec{c}}_{2}^{\varvec{{\prime }}}$)	0.038	0.011	< 0.001	[0.018, 0.060]
Interpersonal needs → Depression				
Total effect	0.151	0.016	< 0.001	[0.120, 0.182]
Indirect effect (paths ${\varvec{a}}_{2}\times {\varvec{b}}_{1}$)	0.101	0.010	< 0.001	[0.083, 0.121]
Direct effect (path ${\varvec{c}}_{4}^{\varvec{{\prime }}}$)	0.050	0.016	0.002	[0.019, 0.082]
Interpersonal needs → Anxiety				
Total effect	0.143	0.016	< 0.001	[0.111, 0.175]
Indirect effect (paths ${\varvec{a}}_{2}\times {\varvec{b}}_{2}$)	0.133	0.013	< 0.001	[0.109, 0.159]
Direct effect (path ${\varvec{c}}_{3}^{\varvec{{\prime }}}$)	0.010	0.017	0.537	[-0.022, 0.044]

***Note.*** S.E. = Standard error, CI = confidence interval. Control variables were age and gender. Paths refer to the hypothesized associations as denoted in Fig. 1

**Fig. 2:**
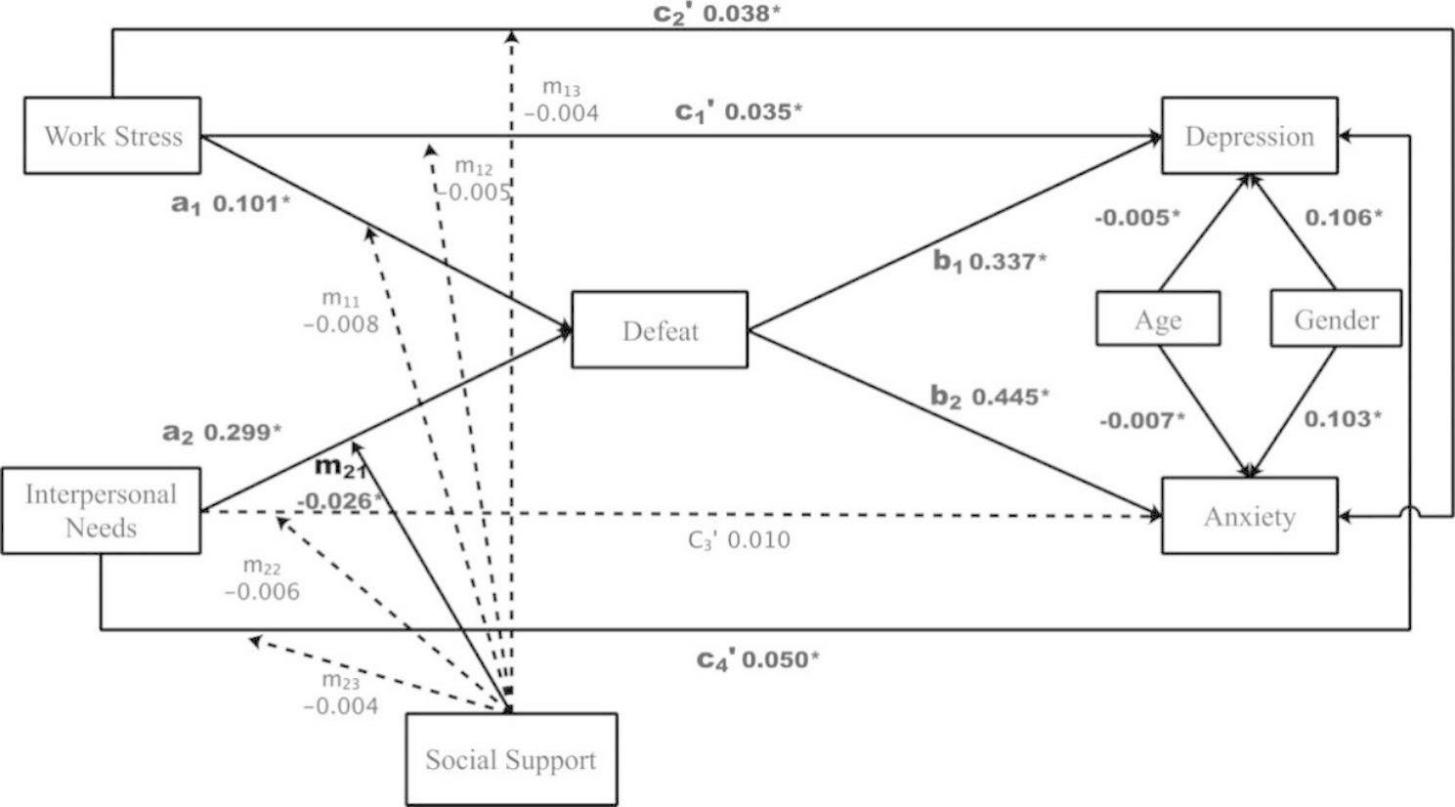
Results of the fitted moderated mediation model ***Note***. * *p* < .05. Significant path coefficients are in bold. Unstandardized coefficients were reported

Another path analysis further examined the proposed moderating role(s) of social support. Model fit was slightly improved by the inclusion of social support and its product terms with work stress and (unmet) interpersonal needs (χ^2^ (2) = 14.072, *p* < .001; RMSEA = 0.055; CFI = 0.995; TLI = 0.940; SRMR = 0.016). Explained variances of the outcome variables were increased well, with the largest increase for defeat (${R}_{defeat}^{2}$= 0.256, ${R}_{depression}^{2}$= 0.255, and ${R}_{anxiety}^{2}$= 0.312; *p*s < 0.001). Results supported Hypotheses 2.2 such that more social support resulted in less anxiety (*B* = -0.015, *SE* = 0.005, *p* = .002, 95% CI = [-0.025, -0.006]) and defeat (*B* = -0.032, *SE* = 0.006, *p* < .001, 95% CI = [-0.043, -0.021]), but not depression (*B* = -0.008, *SE* = 0.005, *p* = .075, 95% CI = [-0.018, 0.001]). Moreover, social support moderated the effect of unmet interpersonal needs on defeat (namely path **m**_**21**_; *B* = -0.026, *SE* = 0.009, *p* = .003, 95% CI = [-0.042, -0.009]), and tentatively the associated indirect effect on depression and anxiety symptoms (namely paths **m**_**21**_ × **b**_**1**_ and **m**_**21**_ × **b**_**2**_), as shown in Fig. 2. More details of this model can be found in Supplementary Table 2.

**Fig. 3:**
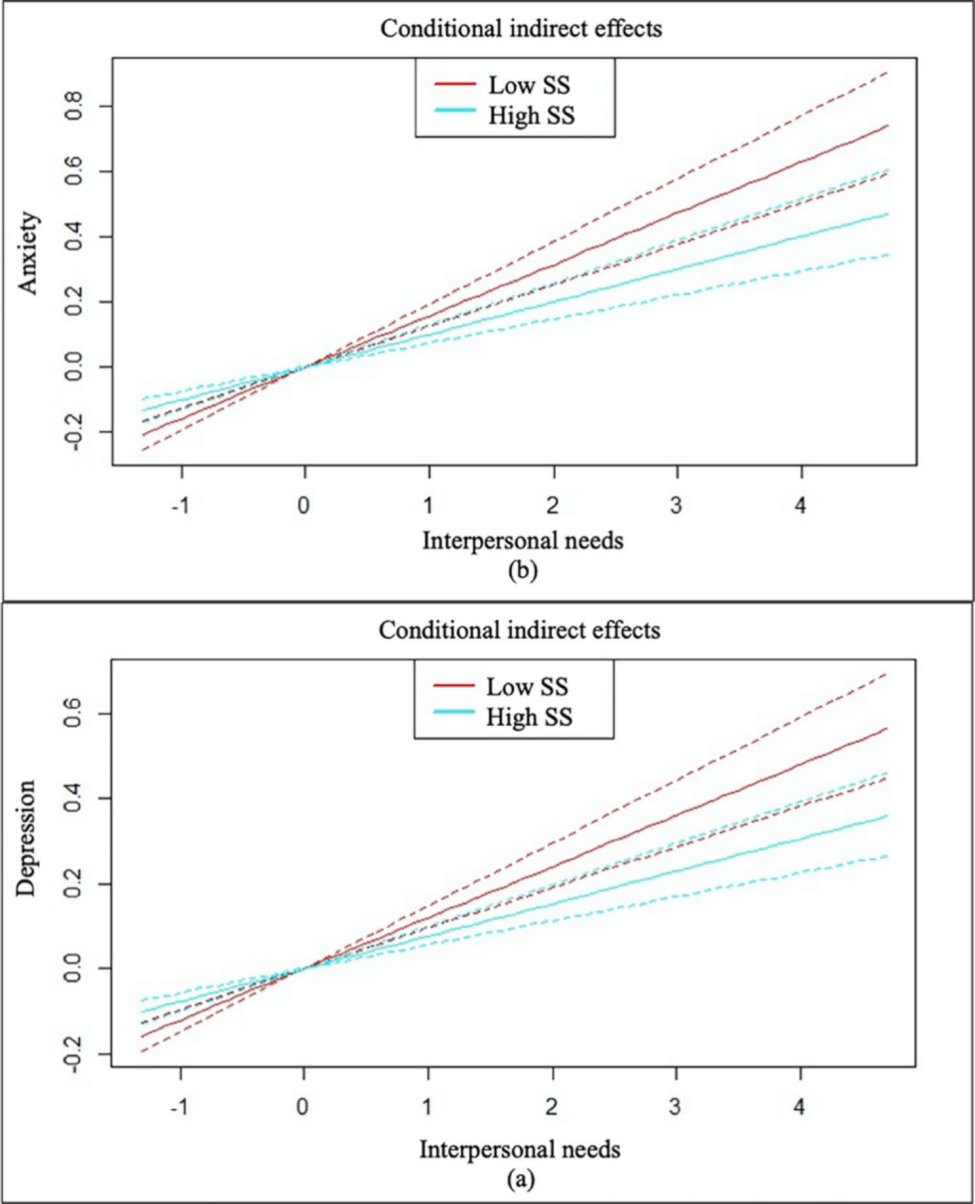
Conditional effects plots ***Note***. Social support (SS) moderated the indirect effects of interpersonal needs via defeat on depression (a) and anxiety (b). Blue and red lines represent conditional effects at higher (i.e., *M* + *SD*) vs. lower (i.e., *M* – *SD*) levels of social support, respectively, while dotted lines represent the upper and lower 95% confidence interval boundaries for corresponding effects

Decomposing the above conditional effects (see Fig. 3), results suggested that those who reported high social support exhibited *fewer* depression symptoms (i.e., *M* + *SD*; *B*_high_ = 0.076, *SE* = 0.011, *p* < .001, 95% CI = [0.056, 0.098]) than their counterparts who reported low social support (i.e., *M* – *SD*; *B*_low_ = 0.120, *SE* = 0.013, *p* < .001, 95% CI = [0.095, 0.148]; *B*_effect difference_ = 0.044, *SE* = 0.015, *p* = .004, 95% CI = [0.015, 0.073]) as a result of unmet interpersonal needs. Similar pattern was found for anxiety (i.e., for *M* + *SD*, *B*_high_ = 0.100, *SE* = 0.014, *p* < .001, 95% CI = [0.074, 0.129]; for *M* – *SD*, *B*_low_ = 0.158, *SE* = 0.017, *p* < .001, 95% CI = [0.126, 0.193]; *B*_effect difference_ = 0.057, *SE* = 0.019, *p* = .003, 95% CI = [0.019, 0.095]).

Finally, a set of supplementary analyses were conducted to probe for differences among industries (i.e., electronic equipment (*n* = 1010), machining (*n* = 356), and other industries (*n* = 641) 1). Results (Supplementary Table 3) revealed that the mediation model hold for participants from electronic equipment and machining factories, except that the defeat *fully* mediated the effects not only from interpersonal needs to anxiety symptoms but also from interpersonal needs to depression symptoms, as well as from work stress to depression and anxiety symptoms in participants from machining factories. Results (Supplementary Table 4) further revealed that the moderating effect of social support on the effect from interpersonal needs to defeat (*B* = -0.048, *SE* = 0.012, *p* < .001, 95% CI = [-0.071, -0.023]), and the associated indirect effects on depression (*B* = 0.304, *SE* = 0.031, *p* < .001, 95% CI = [0.241, 0.363]), and anxiety (*B* = 0.414, *SE* = 0.036, *p* < .001, 95% CI = [0.341, 0.484]) symptoms, were consistent for participants from *electronic equipment* factories. A further decomposition revealed that those who reported high social support exhibited fewerdepression symptom (i.e., *M* + *SD*; *B*_high_ = 0.064, *SE* = 0.015, *p* < .001, 95% CI = [0.037, 0.094]) than their counterparts who reported low social support (i.e., *M* – *SD*; *B*_low_ = 0.139, *SE* = 0.020, *p* < .001, 95% CI = [0.103, 0.181]; *B*_effect difference_ = 0.075, *SE* = 0.022, *p* < .001, 95% CI = [0.034, 0.119]) as a result of unmet interpersonal needs. Similar pattern was found for anxiety (i.e., for *M* + *SD*, *B*_high_ = 0.087, *SE* = 0.021, *p* < .001, 95% CI = [0.047, 0.130]; for *M* – *SD*, *B*_low_ = 0.189, *SE* = 0.024, *p* < .001, 95% CI = [0.145, 0.237]; *B*_effect difference_ = 0.102, *SE* = 0.027, *p* < .001, 95% CI = [0.049, 0.156]). These effects did not hold for participants from machining or the other factories. However, social support moderated the effect of *work stress* on defeat (*B* = -0.033, *SE* = 0.011, *p* = .002, 95% CI = [-0.054, -0.012]), and the associated indirect effects on depression (*B* = 0.315, *SE* = 0.060, *p* < .001, 95% CI = [0.195, 0.427]), and anxiety (*B* = 0.461, *SE* = 0.071, *p* < .001, 95% CI = [0.324, 0.598]) symptoms among participants from machining factories. A further decomposition revealed that the effect difference was significant for depression (*B*_effect difference_ = 0.053, *SE* = 0.019, *p* = .006, 95% CI = [0.021, 0.096]) and anxiety (*B*_effect difference_ = 0.078, *SE* = 0.028, *p* = .005, 95% CI = [0.030, 0.139]).

## Discussion

The present study aimed at examining the mechanisms via which work stress and interpersonal needs casted on Chinese industrial workers’ mental health. Results suggested a high prevalence depression and anxiety symptoms was in concordance with the literatures [56, 57]. Advancing previous studies, it was primarily established the mediating role of defeat in the relationship between work stress and depression/anxiety and the relationship between interpersonal needs and depression/anxiety, as well as the moderating role of social support in a sample of Chinese industrial workers. Specifically, defeat could (partially) account for the work stress and interpersonal needs elicited depression and anxiety symptoms for Chinese industrial workers. Moreover, findings revealed that higher levels of social support were associated with not only fewer anxiety symptoms, but also fewer vulnerabilities to work unsatisfied interpersonal needs associated outcomes.

Supporting *Hypothesis 1.1*, results expanded the (partial) mediation role of defeat in the effect of work stress on depression and anxiety in Chinese industrial workers, a large yet underrepresented population in previous research. On one hand, typically for industrial workers, some compensation systems (e.g., the piece-rate system) could motivate industrial workers to work more to gain more, exacerbating the exposure of work stress in repetitive, mechanical flow line work modes, and limiting the opportunity for positive psychological processes (e.g., self-development, fulfilling autonomy needs). Even so, 70% of the industrial workers gained less than 5,000 RMB per month in the present study, far below the average monthly income of citizens in Shenzhen (about 10,468 RMB per month in 2019) [58]. These processes could result in feelings of defeat, or feelings of failed struggle and low social rank. On the other hand, loss of social rank has been associated with dysregulation of stress relevant psychological [59] and biological responses [60] relevant to depression and anxiety symptoms [21, 26]. This result extended the knowledge of the relationship between work stress and anxiety/depression. Excessive work stress could overwhelm industrial workers to the point where they become unable to cope with competition at work (e.g., promotions), further damaging their mental health.

Supporting *Hypothesis 1.2*, results revealed that defeat fully mediated the effect of thwarted interpersonal needs, but *not* heavy work stress, on depression and anxiety symptoms among industrial workers. Interpersonal needs per se reflect senses of thwarted belongingness and perceived burdensomeness, constitute a risk for severe mental conditions, and predict suicidal behaviours [15]. Nearly 90% of this industrial worker sample were migrants, who had been working migrants in Shenzhen for an average of 6 years. Research on similar populations reported commonly experiencing discrimination and exclusion from local residents, impeding the establishment and maintenance of social bonding [61, 62]. Constant failures of social bonding could naturally serve as a source of defeat, and propagate anxious and depressive symptoms [15]. Together, the support of hypotheses 1.1 and 1.2 substantiate how incompetence in various social arenas confers risks of depression and anxiety via the feelings of defeat [23].

Partially supporting *Hypothesis 2.2*, our results suggested that social support could mitigate the deleterious effect of unmet interpersonal needs on defeat and thus mitigate its associated deleterious effects on depression and anxiety symptoms. These findings were in line with the well-established buffering hypothesis [35]. Notably, the results also supported the main-effect hypothesis such that workers with higher levels of social support reported lower levels of anxiety symptoms [63]. The existence of a supportive social network could affirm one’s values and contribute to personal growth [64], boost subjective social status [65], or facilitate various self-regulation in goal pursuit [66], while the lack thereof constitutes risk conditions for health in general [67]. Therefore, our results here pointed to the prominent role of increasing social support for Chinese industrial workers. Unexpectedly that the moderating effect of social support in the relationship between work stress and depression and anxiety symptoms was not statistically significant. The possible explanation is that considering the closed working environment and mechanical working patterns of industrial workers, social support cannot substantially change their perceived work stress. However, social support can enhance a sense of belonging and reduce feelings of social disconnection and exclusion caused by, for example, closed working environment. Activities providing social interactions can meet their basic psychological needs and make them feel that their existence is meaningful, thus lowering the risk of depression and anxiety [68].

Our major findings were replicated for Chinese industrial workers from electronic equipment and machining factories, but not those from, e.g., dyeing and printing. Even though participants from electronic equipment and machining factories were the majority of the current sample (68.06%), such results might reflect the heterogeneity among workers from different industries. Factors such as working environment, organizational administration, and occupational exposure vary drastically among different industries, which could potentially affect the proposed mechanism. For illustration, compared with the rest categories, workers from electronic equipment and machining factories are exposed to higher risks of physical injury due to their frequent usage of, e.g., electro welding or electric drill, which constitutes a substantial work stress that is rare for industries such as food processing. Such industrial heterogeneity should be taken into consideration when designing interventions.

Taken together, these results suggested the important characteristics of the social environment on stress-coping and associated mental health outcomes for Chinese industrial workers. Not only did it shape the external resources that one could mobilize in stress coping processes, but it also shaped different ways that a stressor might cast on mental health outcomes. To our knowledge, this is the first study to (a) replicate the mediation role of defeat in the relationship between work stress and interpersonal needs and mental disorders in Chinese industrial workers, and (b) to reveal the importance of the conditioning effects of social support. The findings of this study shed new light on improving the mental health of Chinese industrial workers. Organizations can hold regular workshops or training programs to improve industrial workers’ occupational or even social competitiveness in a continuous way to prevent defeat experiences and hence reduce the associated mental health risks. Alternatively, organizations can provide networking events to help industrial workers enrich their social network, and hence the availability of social support when needed.

Apart from its many merits, the cross-sectional nature of this study precluded us from examining the causal relationships between work stress and feelings of defeat, although an alternative model suggests a poor model fit (RMSEA = 0.101; TLI = 0.768). Future studies could benefit from longitudinal designs to gain causality support. Besides, defeat was investigated in isolation from its frequently cooccurring experiences (e.g., entrapment). Future studies could contribute by differentiating the possibly entangled mechanisms of these associated factors and providing a more comprehensive framework of the roles that these negative experiences might play between various stressors and mental health outcomes.

## Conclusion

The current study expanded the mediation role of defeat in the relationship of work stress and interpersonal needs with depression and anxiety symptoms of Chinese industrial workers, a large-scale yet underrepresented population in the field. The current study also revealed the ameliorating role of social support in thwarted interpersonal needs and defeat linkage. The findings shed light on future intervention approaches such as improving perceived social status or providing more self-expansion and self-development opportunities to combat work stress elicited feelings of defeat while cultivating healthy social environments to maximize intervention utilities.

## Electronic supplementary material

Below is the link to the electronic supplementary material.

Supplementary Material 1

## Data Availability

The datasets used during the current study are available from the corresponding author on reasonable request. The data are not publicly available due to protection of participants’ privacy.

## Footnotes

1 Participants from other industries were from dyeing and printing (*n* = 112), raw chemical (*n* = 73), textiles (*n* = 23), food processing (*n* = 24), melting (*n* = 8), and those who did not report their industrials (*n* = 401). The relatively limited sample sizes in these sub-categories led to model convergence issues and were therefore combined.
