# Supplementary material for: Relationships of work stress and interpersonal needs with industrial workers’ mental health: a moderated mediation model
Source: BMC Public Health. 2023 Jul 12;23:1341. doi: 10.1186/s12889-023-16002-1 (PMC10339573; doi:10.1186/s12889-023-16002-1)
Supplement: Supplementary file 1 — Supplementary Material 1 [file 12889_2023_16002_MOESM1_ESM.docx]

**Supplementary Table 1**

Sample sociodemographic characteristics (*n* = 2007).

| Sociodemographic Characteristic | *M* ± *SD*/ Count (%) |
| --- | --- |
| **Age (years)** | 33.01 ± 7.92 |
| **Gender** |  |
| Male | 1352 (67.4) |
| Female | 655 (32.6) |
| **Marital status** |  |
| Unmarried | 795 (39.6) |
| Married | 1081 (53.9) |
| Divorced/Widowed | 51 (2.5) |
| Unknown | 80 (4.0) |
| **Education level** |  |
| Below high school | 1181 (58.9) |
| High school | 644 (32.1) |
| College degree or above | 124 (6.1) |
| Unknown | 58 (2.9) |
| **Income (Chinese yuan)** |  |
| < 3000 | 182 (9.0) |
| 3000 - 4999 | 1230 (61.3) |
| > 4999 | 526 (26.2) |
| Unknown | 69 (3.4) |
| **Number of Child** |  |
| 0 | 895 (44.6) |
| 1-2 | 982 (49.0) |
| 3-5 | 130 (6.4) |
| **Place of origin** |  |
| Shenzhen | 29 (1.4) |
| Out of Shenzhen | 1792(89.3) |
| Unknown | 186 (9.3) |
| **Live alone or not** |  |
| Yes | 659 (32.8) |
| No | 1348 (67.2) |
| **Weekly working time (hours)** | 46.73 ± 17.52 |
| **Duration participants worked as migrants in Shenzhen (years)** | 6.24 ± 6.33 |

*Note*. *M* = mean. *SD* = standard deviation.

**Supplementary Table 2**

Results of the moderated mediation model (N=2007).

| (Outcome) | Estimate | *S.E.* | *p* | Bootstrap 95% CI |
| --- | --- | --- | --- | --- |
| **Depression** |  |  |  |  |
| Work stress | **0.032** | **0.011** | **.003** | **[0.011, 0.053]** |
| Interpersonal needs | **0.053** | **0.016** | **.001** | **[0.021, 0.085]** |
| Defeat | **0.330** | **0.022** | **< .001** | **[0.286, 0.372]** |
| Social support | -0.008 | 0.005 | .075 | [-0.018, 0.001] |
| Work stress × Social support | -0.005 | 0.004 | .263 | [-0.014, 0.003] |
| Interpersonal needs × Social support | -0.004 | 0.006 | .527 | [-0.016, -0.008] |
| **Anxiety** |  |  |  |  |
| Work stress | **0.035** | **0.010** | **.001** | **[0.015, 0.056]** |
| Interpersonal needs | 0.015 | 0.017 | .395 | [-0.019, 0.048] |
| Defeat | **0.433** | **0.025** | **<.001** | **[0.382, 0.481]** |
| Social support | **-0.015** | **0.005** | **.002** | **[-0.025, -0.006]** |
| Work stress × Social support | -0.004 | 0.004 | .266 | [-0.013, 0.003] |
| Interpersonal needs × Social support | -0.006 | 0.006 | .307 | [-0.016, 0.005] |
| **Defeat (Mediator)** |  |  |  |  |
| Work stress | **0.091** | **0.014** | **< .001** | **[0.063, 0.118]** |
| Interpersonal needs | **0.298** | **0.022** | **< .001** | **[0.254, 0.342]** |
| Social support | **-0.032** | **0.006** | **< .001** | **[-0.043, -0.021]** |
| Work stress × Social support | -0.008 | 0.006 | .193 | [-0.019, 0.004] |
| Interpersonal needs × Social support | **-0.026** | **0.009** | **.003** | **[-0.042, -0.009]** |

***Note.*** *S.E.* = standard error. CI = bootstrapping confidence interval. Control variables were age and gender.

**Supplementary Table 3**

Comparing the mediating effect(s) of defeat across different industrials.

|  | Electronic equipment (*n* = 1010) | | | |  | Machining (*n* = 356) | | | |  | Other (*n* = 641) | | | |
| --- | --- | --- | --- | --- | --- | --- | --- | --- | --- | --- | --- | --- | --- | --- |
| (Outcome) | Estimate | *S.E.* | *p* | Bootstrap 95% CI |  | Estimate | *S.E.* | *p* | Bootstrap 95% CI |  | Estimate | *S.E.* | *p* | Bootstrap 95% CI |
| Work stress → Depression |  |  |  |  |  |  |  |  |  |  |  |  |  |  |
| Total effect | 0.079 | 0.018 | < .001 | [0.044, 0.114] |  | 0.058 | 0.023 | .010 | [0.014, 0.103] |  | 0.224 | 1.237 | .856 | [-2.150, 2.696] |
| Indirect effect (paths $\boldsymbol{a}_{\boldsymbol{1}}\text{×}\text{ }\text{b}_{\text{1}}$) | 0.036 | 0.007 | < .001 | [0.024, 0.051] |  | 0.034 | 0.010 | .001 | [0.016, 0.056] |  | -0.139 | 0.338 | .681 | [-0.795, 0.550] |
| Direct effect (path $\boldsymbol{c}_{\boldsymbol{1}}^{\boldsymbol{'}}$) | 0.045 | 0.017 | .011 | [0.010, 0.076] |  | 0.025 | 0.023 | .281 | [-0.022, 0.068] |  | 0.364 | 1.151 | .752 | [-1.834, 2.662] |
| Work stress → Anxiety |  |  |  |  |  |  |  |  |  |  |  |  |  |  |
| Total effect | 0.115 | 0.017 | < .001 | [0.082, 0.149] |  | 0.044 | 0.025 | .072 | [-0.001, 0.096] |  | -0.016 | 0.021 | .452 | [-0.049, 0.035] |
| Indirect effect (paths $\boldsymbol{a}_{\boldsymbol{1}}\times\boldsymbol{b}_{\boldsymbol{2}}$) | 0.049 | 0.010 | < .001 | [0.032, 0.070] |  | 0.050 | 0.015 | .001 | [0.023, 0.084] |  | 0.001 | 0.003 | .696 | [-0.004, 0.007] |
| Direct effect (path $\boldsymbol{c}_{\boldsymbol{2}}^{\boldsymbol{'}}$) | 0.066 | 0.016 | < .001 | [0.036, 0.097] |  | -0.005 | 0.025 | .834 | [-0.053, 0.043] |  | -0.017 | 0.021 | .421 | [-0.050, 0.034] |
| Interpersonal needs → Depression |  |  |  |  |  |  |  |  |  |  |  |  |  |  |
| Total effect | 0.201 | 0.023 | < .001 | [0.155, 0.244] |  | 0.154 | 0.032 | < .001 | [0.094, 0.218] |  | 2.768 | 0.634 | < .001 | [1.567, 4.043] |
| Indirect effect (paths $\boldsymbol{a}_{\boldsymbol{2}}\times\boldsymbol{b}_{\boldsymbol{1}}$) | 0.107 | 0.014 | < .001 | [0.082, 0.137] |  | 0.098 | 0.027 | < .001 | [0.041, 0.155] |  | 2.759 | 0.424 | < .001 | [2.006, 3.693] |
| Direct effect (path $\boldsymbol{c}_{\boldsymbol{4}}^{\boldsymbol{'}}$) | 0.094 | 0.025 | < .001 | [0.044, 0.141] |  | 0.056 | 0.039 | .146 | [-0.019, 0.120] |  | 0.008 | 0.697 | .991 | [-1.283, 1.447] |
| Interpersonal needs → Anxiety |  |  |  |  |  |  |  |  |  |  |  |  |  |  |
| Total effect | 0.168 | 0.024 | < .001 | [0.122, 0.214] |  | 0.146 | 0.040 | < .001 | [0.068, 0.223] |  | -0.035 | 0.009 | < .001 | [-0.056, -0.019] |
| Indirect effect (paths $\boldsymbol{a}_{\boldsymbol{2}}\times\boldsymbol{b}_{\boldsymbol{2}}$) | 0.146 | 0.019 | < .001 | [0.111, 0.184] |  | 0.145 | 0.037 | < .001 | [0.079, 0.223] |  | -0.021 | 0.007 | .003 | [-0.036, -0.009] |
| Direct effect (path $\boldsymbol{c}_{\boldsymbol{3}}^{\boldsymbol{'}}$) | 0.022 | 0.027 | .424 | [-0.030, 0.075] |  | 0.001 | 0.049 | .986 | [-0.096, 0.093] |  | -0.014 | 0.006 | .018 | [-0.028, -0.004] |

***Note***. *S.E.* = standard error. CI = confidence interval. Control variables were age and gender.

**Supplementary Table 4**

Comparing the moderating effect(s) of social support across different industrials

|  | Electronic equipment (*n* = 1010) | | | |  | Machining (*n* = 356) | | | |  | Other (*n* = 641) | | | |
| --- | --- | --- | --- | --- | --- | --- | --- | --- | --- | --- | --- | --- | --- | --- |
| (Outcome) | Estimate | *S.E.* | *p* | Bootstrap 95% CI |  | Estimate | *S.E.* | *p* | Bootstrap 95% CI |  | Estimate | *S.E.* | *p* | Bootstrap 95% CI |
| **Depression** |  |  |  |  |  |  |  |  |  |  |  |  |  |  |
| Work stress | 0.043 | 0.016 | .009 | [0.011, 0.075] |  | 0.026 | 0.023 | .255 | [-0.021, 0.067] |  | -0.017 | 0.021 | .428 | [-0.050, 0.035] |
| Interpersonal needs | 0.102 | 0.023 | < .001 | [0.054, 0.146] |  | 0.060 | 0.038 | .117 | [-0.013, 0.134] |  | -0.040 | 0.012 | .001 | [-0.063, -0.015] |
| Defeat | 0.304 | 0.031 | < .001 | [0.241, 0.363] |  | 0.315 | 0.060 | < .001 | [0.195, 0.427] |  | -0.025 | 0.007 | .001 | [-0.041, -0.012] |
| Social support | -0.008 | 0.007 | .252 | [-0.022, 0.005] |  | -0.017 | 0.012 | .153 | [-0.041, 0.007] |  | -0.001 | 0.001 | .327 | [-0.002, 0.001] |
| Work stress × Social support | -0.010 | 0.007 | .172 | [-0.026, 0.003] |  | -0.005 | 0.010 | .647 | [-0.024, 0.016] |  | 0.002 | 0.001 | .153 | [-0.001, 0.005] |
| Interpersonal needs × Social support | -0.015 | 0.011 | .162 | [-0.036, 0.006] |  | 0.025 | 0.011 | .019 | [0.004, 0.046] |  | 0.002 | 0.001 | .078 | [0.000, 0.004] |
| **Anxiety** |  |  |  |  |  |  |  |  |  |  |  |  |  |  |
| Work stress | 0.064 | 0.015 | < .001 | [0.035, 0.093] |  | -0.006 | 0.025 | .811 | [-0.056, 0.043] |  | -0.013 | 0.054 | .806 | [-0.118, 0.092] |
| Interpersonal needs | 0.033 | 0.026 | .213 | [-0.018, 0.002] |  | 0.007 | 0.048 | .885 | [-0.082, 0.101] |  | -0.155 | 0.040 | < .001 | [-0.230, -0.075] |
| Defeat | 0.414 | 0.036 | <.001 | [0.341, 0.484] |  | 0.461 | 0.071 | <.001 | [0.324, 0.598] |  | 0.370 | 0.053 | < .001 | [0.263, 0.473] |
| Social support | -0.022 | 0.007 | .001 | [-0.036, -0.009] |  | -0.022 | 0.013 | .080 | [-0.048, 0.001] |  | 0.013 | 0.002 | < .001 | [0.010, 0.017] |
| Work stress × Social support | -0.008 | 0.007 | .219 | [-0.023, 0.003] |  | -0.000 | 0.011 | .965 | [-0.022, 0.022] |  | 0.004 | 0.004 | .317 | [-0.004, 0.011] |
| Interpersonal needs × Social support | -0.017 | 0.009 | .046 | [-0.033, 0.002] |  | 0.016 | 0.014 | .238 | [-0.010, 0.043] |  | -0.007 | 0.003 | .024 | [-0.013, -0.001] |
| **Defeat (Mediator)** |  |  |  |  |  |  |  |  |  |  |  |  |  |  |
| Work stress | 0.107 | 0.019 | < .001 | [0.069, 0.146] |  | 0.093 | 0.033 | .005 | [0.038, 0.169] |  | -0.090 | 0.031 | .004 | [-0.145, -0.023] |
| Interpersonal needs | 0.333 | 0.030 | < .001 | [0.274, 0.392] |  | 0.296 | 0.046 | < .001 | [0.201, 0.381] |  | 0.075 | 0.038 | .046 | [0.003, 0.152] |
| Social support | -0.035 | 0.008 | < .001 | [-0.050, -0.018] |  | -0.065 | 0.013 | < .001 | [-0.091, -0.039] |  | 0.005 | 0.002 | < .001 | [0.005, 0.008] |
| Work stress × Social support | -0.006 | 0.009 | .485 | [-0.023, 0.011] |  | -0.033 | 0.011 | .002 | [-0.054, -0.012] |  | 0.007 | 0.001 | .007 | [-0.008, -0.001] |
| Interpersonal needs × Social support | -0.048 | 0.012 | < .001 | [-0.071, -0.023] |  | -0.016 | 0.017 | .339 | [-0.048, 0.017] |  | -0.005 | 0.002 | .063 | [0.000, 0.010] |

***Note.*** *S.E.* = standard error. CI = bootstrapping confidence interval. Control variables were age and gender.
